# Added value of 3T MRI and the MRI-halo sign in assessing resectability of locally advanced pancreatic cancer following induction chemotherapy (IMAGE-MRI): prospective pilot study

**DOI:** 10.1007/s00423-022-02653-y

**Published:** 2022-10-15

**Authors:** Thomas F. Stoop, Eran van Veldhuisen, L. Bengt van Rijssen, Remy Klaassen, Oliver J. Gurney-Champion, Ignace H. de Hingh, Olivier R. Busch, Hanneke W. M. van Laarhoven, Krijn P. van Lienden, Jaap Stoker, Johanna W. Wilmink, C. Yung Nio, Aart J. Nederveen, Marc R. W. Engelbrecht, Marc G. Besselink, IMAGE study group, Koop Bosscha, Loes van den Nieuwehof-Biesheuvel, Hendrik A. Marsman, Leonard W. F. Seelen

**Affiliations:** 1 Amsterdam UMC, location University of Amsterdam, Department of Surgery, Amsterdam, The Netherlands; 2 Cancer Center, Amsterdam, The Netherlands; 3 Amsterdam UMC, location University of Amsterdam, Department of Medical Oncology, Amsterdam, The Netherlands; 4 Amsterdam UMC, location University of Amsterdam, Department of Radiology and Nuclear Medicine, Amsterdam, The Netherlands; 5 Department of Surgery, Catharina Hospital Eindhoven, Eindhoven, the Netherlands; 6 Department of Radiology, St Antonius Hospital Nieuwegein, University Medical Center Utrecht Cancer Center, Regional Academic Cancer Center Utrecht, Nieuwegein, the Netherlands

**Keywords:** Locally advanced pancreatic cancer, Induction chemotherapy, Staging, Resectability

## Abstract

**Background:**

Restaging of locally advanced pancreatic cancer (LAPC) after induction chemotherapy using contrast-enhanced computed tomography (CE-CT) imaging is imprecise in evaluating local tumor response. This study explored the value of 3 Tesla (3 T) contrast-enhanced (CE) and diffusion-weighted (DWI) magnetic resonance imaging (MRI) for local tumor restaging.

**Methods:**

This is a prospective pilot study including 20 consecutive patients with LAPC with RECIST non-progressive disease on CE-CT after induction chemotherapy. Restaging CE-CT, CE-MRI, and DWI-MRI were retrospectively evaluated by two abdominal radiologists in consensus, scoring tumor size and vascular involvement. A halo sign was defined as replacement of solid perivascular (arterial and venous) tumor tissue by a zone of fatty-like signal intensity.

**Results:**

Adequate MRI was obtained in 19 patients with LAPC after induction chemotherapy. Tumor diameter was non-significantly smaller on CE-MRI compared to CE-CT (26 mm vs. 30 mm; *p* = 0.073). An MRI-halo sign was seen on CE-MRI in 52.6% (*n* = 10/19), whereas a CT-halo sign was seen in 10.5% (*n* = 2/19) of patients (*p* = 0.016). An MRI-halo sign was not associated with resection rate (60.0% vs. 62.5%; *p* = 1.000). In the resection cohort, patients with an MRI-halo sign had a non-significant increased R0 resection rate as compared to patients without an MRI-halo sign (66.7% vs. 20.0%; *p* = 0.242). Positive and negative predictive values of the CE-MRI-halo sign for R0 resection were 66.7% and 66.7%, respectively.

**Conclusions:**

3 T CE-MRI and the MRI-halo sign might be helpful to assess the effect of induction chemotherapy in patients with LAPC, but its diagnostic accuracy has to be evaluated in larger series.

**Supplementary information:**

The online version contains supplementary material available at 10.1007/s00423-022-02653-y.

## Introduction

Approximately 30–40% of the patients with pancreatic ductal adenocarcinoma (PDAC) present with locally advanced pancreatic cancer (LAPC) [1]. LAPC is characterized by extensive vascular tumor involvement prohibiting an upfront curative-intent resection [2]. The introduction of FOLFIRINOX (i.e., a combination of 5-fluorouracil, leucovorin, irinotecan, and oxaliplatin) and gemcitabine-nab-paclitaxel induction chemotherapy has resulted in the opportunity to achieve local and systemic control of LAPC. As a consequence, a resection is possible in 16–26% of patients with LAPC [3–5], associated with survival rates comparable to those obtained in (borderline) resectable disease [6–9].

Patient selection for surgical exploration is based on both tumor biology [10] and anatomical staging [11, 12]. Unfortunately, contrast-enhanced computed tomography (CE-CT) imaging is inaccurate to determine the resectability of LAPC following induction therapy because it cannot differentiate between vital tumor tissue and fibrosis [13], whereby the sensitivity and specificity on the resectability in patients with PDAC who underwent resection after preoperative chemo(radio)therapy are 45–78% and 60–85%, respectively [14, 15]. Consequently, local response to chemotherapy may be underestimated, which might result in missed opportunities for resection or, conversely, futile exploratory laparotomy [16]. Improvement of preoperative imaging modalities is urgently needed to reduce futile surgery and prevent the associated poor short- and long-term outcomes [17].

In pancreatic cancer, contrast-enhanced magnetic resonance imaging (CE-MRI) is generally recommended only as additional imaging next to CE-CT in case of a poorly visible tumor and to exclude liver metastases [18, 19]. Diffusion-weighted magnetic resonance imaging (DWI-MRI) has shown promising results in determining tumor response after chemotherapy in PDAC, focusing on the apparent diffusion coefficient (ADC) and its possible correlation with histological tumor response [20–22]. However, the role of CE-MRI and DWI-MRI for morphological response evaluation after chemotherapy is poorly investigated to our knowledge [16, 23, 24], especially when focusing on the perivascular zone of the tumor to depict a “halo sign,” which is considered to reflect tumor regression [25, 26]. A halo sign has been studied and described with CE-CT [25, 26], but not with MRI. A halo sign could be a valuable marker for remaining vascular tumor involvement after induction chemotherapy and hereby could improve the preoperative anatomical staging and subsequent patient selection for surgery [19, 25–27]. As other studies have shown that DWI-MRI can differentiate vital tumor tissue from fibrosis in rectal cancer after chemoradiation therapy and CE-MRI is assumed to differentiate better between different tissue types [28], we hypothesized this may also be the case in patients with PDAC.

Therefore, this prospective pilot study explored the value of CE-MRI and DWI-MRI separately, compared with standard CE-CT to determine tumor size and vascular involvement of LAPC following induction chemotherapy.

## Materials and methods

This prospective observational pilot study was performed following the Strengthening the Reporting of Observational Studies in Epidemiology (STROBE) statement guideline [29].

The Medical Ethics Committee from the Amsterdam UMC (location Academic Medical Center) approved this prospective study (registration number: NL5665.018.16). Before inclusion, all patients provided written informed consent.

Primary endpoints included tumor size and vascular tumor involvement after induction chemotherapy on CE-CT *versus* CE-MRI and CE-CT *versus* DWI-MRI, including the presence of a halo sign. The secondary endpoints included subjective image quality of CE-CT *versus* CE-MRI and CE-CT *versus* DWI-MRI.

### Study population

The IMAGE-MRI study was conducted between October 2016 and October 2018 and included 20 patients with histologically-proven LAPC at diagnosis and thereafter treated with at least 2 months of induction chemotherapy followed by surgical exploration with the intention for resection. After completing the 2 months of induction chemotherapy, all patients underwent a single 3 Tesla (3 T) MRI including CE-T1-weighted imaging and DWI before surgical exploration, in addition to routine CE-CT scan assessment.

All surgical procedures were performed in three high-volume pancreatic cancer centers, performing at least 40 pancreatoduodenectomies annually. Patients were considered ineligible for inclusion in case of (1) progressive disease on CT scan after induction chemotherapy in accordance with the Response Evaluation Criteria In Solid Tumors (RECIST) definition [30]; (2) metal implants incompatible with MRI imaging; (3) severe claustrophobia prohibiting MRI scan; or (4) impaired renal function prohibiting the administration of intravenous gadolinium contrast.

### Study procedures

Only CT scans were used for clinical decision-making, including patient selection for surgical exploration. The pancreatic surgeons were blinded for the outcomes of the MRI scans, so the findings on MRI were not used for clinical decision-making. See Supplementary Digital Content 1 for procedural details of the CT scans and MRI.

### Classification

Tumor characteristics including largest tumor diameter (axial plane) and vascular tumor involvement on CE-CT scan and MRI were scored with predefined scoring forms in consensus by two abdominal radiologists experienced in pancreatic imaging (M.R.W.E. and C.Y.N., both > 15 years of experience). The radiologists scored the subjective image quality, using a four-point scale: excellent, moderate, poor (clinically unusable/inappropriate), and invisible (clinically unusable/inappropriate). Identification and delineation of the pancreatic tumor as well as the visibility of vascular involvement were scored using this scale.

Comparing the restaging CE-CT scans and MRI after induction therapy, a difference in vascular tumor involvement was defined as a change between the following categories: 0°, 1–90°, 91–180°, 181–270°, > 270°. In the case of portomesenteric venous involvement, the length of tumor involvement in millimeters was used if the degree of involvement was similar on CT scan and MRI. This strategy to use of the degrees and length of vascular tumor involvement is derived from the classification from Ahmed and colleagues [31]. If multiple major vessels were involved whereby one vessel showed more involvement on one of the imaging modalities whereas the other vessel had less involvement, the number of vessels and the length of portomesenteric venous involvement were used for the categorization into *decreased*, *stable*, and *increased* vascular involvement.

### Definitions

LAPC was defined at time of diagnosis (so before the start of systemic treatment) as > 90° arterial contact (i.e., superior mesenteric artery, celiac axis, and/or any hepatic artery) and/or > 270° portomesenteric venous involvement or occlusion [32, 33]. In addition, patients with non-resectable tumors based on solely multivisceral involvement, vascular involvement according to intraoperative findings during exploratory laparotomy, and physical performance status were also classified as LAPC.

The tumor markers carbohydrate antigen 19–9 (CA 19–9) and carcinoembryonic antigen (CEA) were considered elevated when the serum levels exceeded the cut-offs of ≥ 37 U/ml for CA 19–9 and ≥ 5 ng/ml for CEA, respectively. The type of pancreatectomy and its extent were defined in accordance with the International Study Group on Pancreatic Surgery definition [34]. Pancreatic adenocarcinoma was defined following the World Health Organization definition [35]. Microscopic negative residual tumor margin (R0) was defined as a tumor-free resection margin of at least 1 mm in all directions, following the Royal College of Pathologists definition [36]. Tumor within 1 mm from the anterior margin was not defined as R1.

**Fig. 1:**
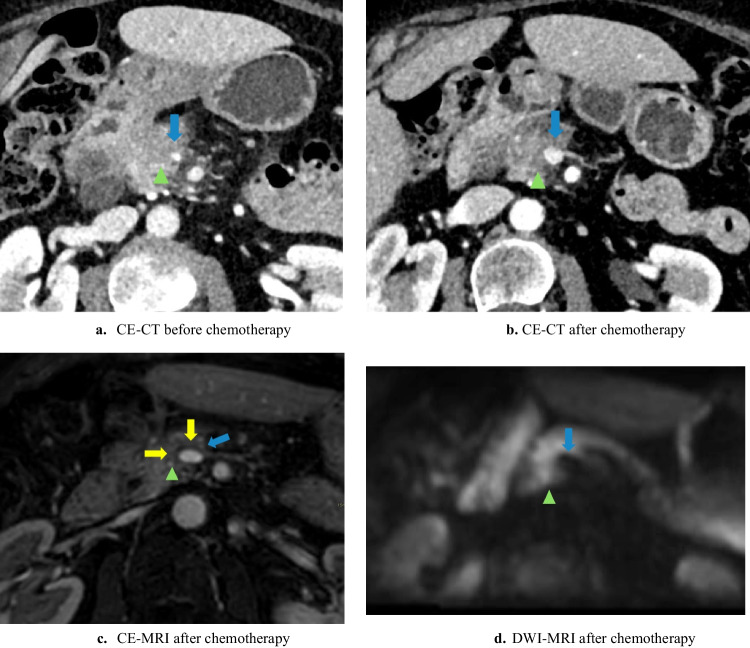
Halo sign on CE-MRI

**Fig. 2:**
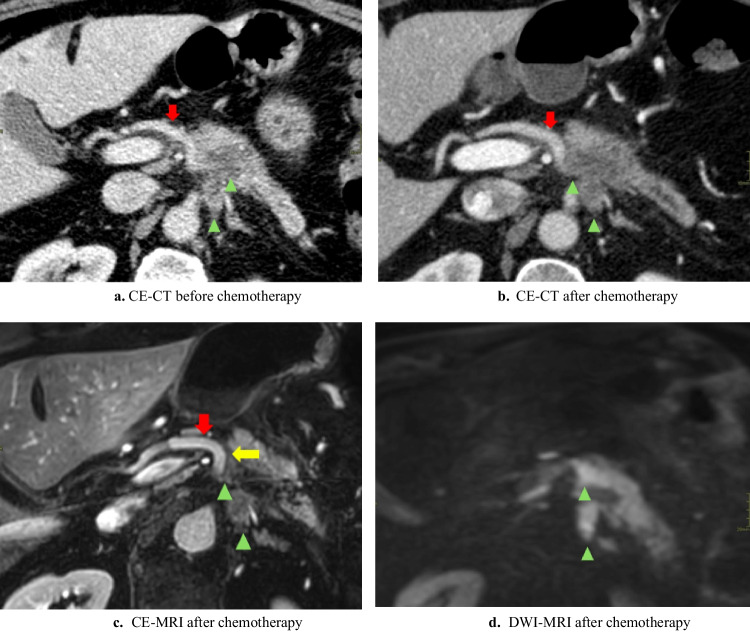
Halo sign on CE-MRI

**Fig. 3:**
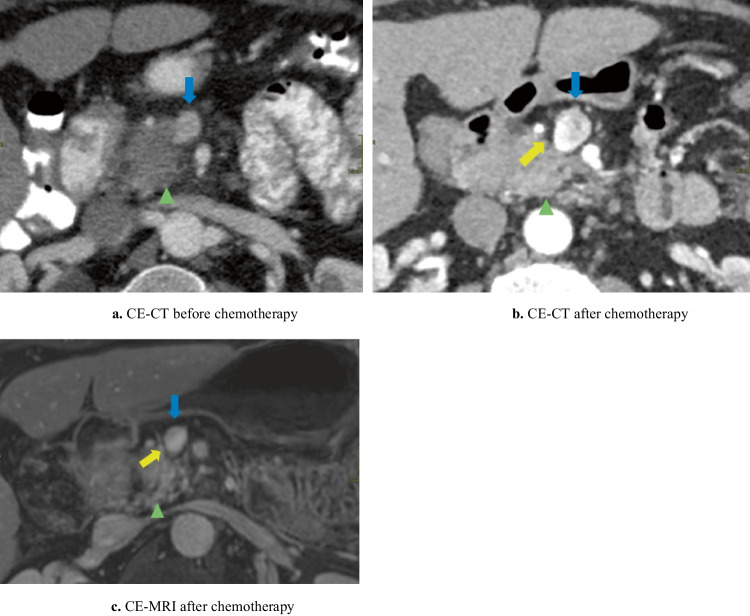
Halo sign on CE-MRI and CE-CT

The MRI-halo sign was defined as partial or complete replacement of solid perivascular tumor tissue by a perivascular (i.e., arterial and venous) zone of signal intensity with fat as proposed for CE-CT imaging and now used similarly for MRI [23, 25] (see Figs. 1c, 2c, and 3c).

### Statistical analysis

Statistical analyses were performed using IBM SPSS Statistics for Windows version 26.0 (IBM Corp., Orchard Road Armonk, NY, USA). Continuous data with a non-normal distribution were presented as medians and interquartile ranges (IQR), and normally distributed continuous data were presented as mean with a standard deviation. Unpaired comparisons were analyzed with the Mann–Whitney *U* test, whereas the Wilcoxon signed rank test was used for paired analyses. Categorical data are presented as frequencies and percentages and compared using Pearson’s chi-square test or Fisher’s exact test when appropriate (unpaired analyses) or McNemar’s test (paired analyses). A two-tailed *p* value < 0.050 was considered statistically significant.

## Results

Out of 22 eligible patients, two patients were not able to undergo an MRI because of claustrophobia, leading to a final cohort of 20 patients. Except one patient, all MRI scans were performed after a CE-CT scan. The mean time interval between CE-CT and MRI was 25 days (± 14).

### Baseline characteristics

A total of 20 patients were included, comprising 9 male (45.0%) and 11 female (55.0%) patients with a median age of 65 years (IQR, 54–67). CA 19–9 was determined in 18/20 patients; median of 357 U/ml (IQR, 156–1559) with elevated levels in 88.9% of patients (*n* = 16/18). CEA was determined in 8/20 patients, comprising a median measured value of 4.2 ng/ml (IQR 3.0–9.4) from which 37.5% (*n* = 3/8) of patients had elevated levels.

All tumors were anatomically staged with CE-CT at the time of diagnosis, except for one patient for whom an MRI was performed. The median tumor size was 37 mm (IQR, 33–44), mostly located in the pancreatic head (*n* = 13/20, 65.0%). Deviated arterial anatomy was present in 45.0% of patients (*n* = 9/20).

The majority of patients (*n* = 15/20, 75.0%) was classified as LAPC based on the degree of venous and/or arterial tumor involvement. The remaining patients were considered LAPC, based on multivisceral tumor involvement (*n* = 2/20, 10.0%), portomesenteric venous involvement according to intraoperative findings elsewhere (*n* = 1/20, 5.0%), length and/or stenosis of portomesenteric venous axis (*n* = 1/20, 5.0%), or physical performance status (*n* = 1/20, 5.0%).

### Induction therapy

All patients were treated with induction chemotherapy; 95.0% (*n* = 19/20) of patients received (m)FOLFIRINOX with a median of 4 courses (IQR, 4–5) and one patient was treated with 3 courses of gemcitabine-nab-paclitaxel.

### Restaging

CA 19–9 levels were elevated after induction chemotherapy in 84.2% of patients (*n* = 16/19); median of 115 U/ml (IQR, 55–596). From the patients with elevated CA 19–9 before induction chemotherapy (*n* = 16) in whom CA 19–9 was also measured after induction chemotherapy (*n* = 15), a ≥ 30% CA 19–9 reduction [37] was seen in *n* = 9/15 (60.0%) patients, but normalization did not occur in any of the patients.

CEA levels were measured in 14 patients; median of 4.8 ng/ml (IQR, 2.4–9.3), and the levels were elevated in 7 patients. Among the patients with elevated CEA before induction chemotherapy (*n* = 3), normalization occurred in *n* = 1/3 (33.3%) patient.

#### CE-CT—staging versus restaging

When comparing the CE-CT scans before and after induction chemotherapy, the patient who was initially staged with MRI was excluded for this comparison. The visibility of vascular involvement before chemotherapy was defined as *moderate/excellent* in the majority of cases (*n* = 18/19, 94.7%), being somewhat less at restaging (*n* = 15/19, 78.9%) (*p* = 0.250).

On restaging, the median tumor size was smaller in comparison to the size at time of diagnosis (37 mm, IQR 34–44 *vs.* 30 mm, IQR 26–42; *p* = 0.023). See Table 1 for the degree of vascular involvement before and after induction chemotherapy and see Table 2 for the details per patient.

**Table 1:** Staging and restaging imaging

	Staging	Restaging		
	CE-CT*	CE-CT	CE-MRI	DWI-MRI
Tumor size (mm), median (IQR)	37 (33–44)	30 (26–42)	27 (21–38)	28 (22–32)
Not assessable, *n* (%)	0 (0)	1 (5.0)	2 (10.0)	5 (25.0)
Arterial involvement				
Superior mesenteric artery, *n* (%)				
No contact	11 (55.0)	11 (55.0)	14 (70.0)	5 (25.0)
1–90°	2 (10.0)	4 (20.0)	1 (5.0)	0 (0)
91–180°	6 (30.0)	4 (20.0)	3 (15.0)	0 (0)
181–270°	1 (5.0)	0 (0)	0 (0)	0 (0)
> 270°	0 (0)	1 (5.0)	1 (5.0)	0 (0)
Not assessable	0 (0)	0 (0)	1 (5.0)	15 (75.0)
Celiac axis, *n* (%)				
No contact	16 (80.0)	16 (80.0)	17 (75.0)	4 (20.0)
1–90°	1 (5.0)	2 (10.0)	0 (0)	0 (0)
91–180°	2 (10.0)	1 (5.0)	1 (5.0)	0 (0)
181–270°	0 (0)	0 (0)	0 (0)	0 (0)
> 270°	1 (5.0)	1 (5.0)	1 (5.0)	0 (0)
Not assessable	0 (0)	0 (0)	1 (5.0)	16 (80.0)
Hepatic artery, *n* (%)				
No contact	12 (60.0)	12 (60.0)	14 (70.0)	4 (20.0)
1–90°	0 (0)	1 (5.0)	0 (0)	0 (0)
91–180°	6 (30.0)	4 (20.0)	2 (10.0)	0 (0)
181–270°	0 (0)	0 (0)	1 (5.0)	0 (0)
> 270°	2 (10.0)	2 (10.0)	2 (10.0)	0 (0)
Not assessable	0 (0)	1 (5.0)	1 (5.0)	16 (80.0)
Portomesenteric venous involvement				
Portal vein, *n* (%)				
No contact	10 (50.0)	13 (65.0)	11 (55.0)	5 (25.0)
1–90°	4 (20.0)	2 (10.0)	3 (15.0)	0 (0)
91–180°	4 (20.0)	4 (20.0)	2 (10.0)	0 (0)
181–270°	0 (0)	0 (0)	0 (0)	0 (0)
> 270°	2 (10.0)	1 (5.0)	1 (5.0)	0 (0)
Not assessable	0 (0)	0 (0)	3 (15.0)	16 (80.0)
Superior mesenteric vein, *n* (%)				
No contact	7 (35.0)	7 (35.0)	13 (65.0)	5 (25.0)
1–90°	2 (10.0)	3 (15.0)	2 (10.0)	0 (0)
91–180°	7 (35.0)	7 (35.0)	2 (10.0)	1 (5.0)
181–270°	0 (0)	0 (0)	0 (0)	0 (0)
> 270°	4 (20.0)	2 (10.0)	1 (5.0)	0 (0)
Not assessable	0 (0)	1 (5.0)	2 (10.0)	14 (70.0)

*CE-CT*, contrast-enhanced computed tomography; *CE-MRI*, contrast-enhanced magnetic resonance imaging; *DWI-MRI*, diffusion-weighted imaging magnetic resonance imaging; *mm*, millimeters; *U/A*, unassessable; *staging MRI instead of CE-CT (*n* = 1); *n*, number of patients; *IQR*, interquartile range. Patients in which the vascular tumor involvement was not assessable on (one of the) imaging modalities are also included in the nominator to illustrate the usefulness of each imaging modality

**Table 2:** Vascular tumor involvement

	SUPERIOR MESENTERIC ARTERY				CELIAC AXIS				HEPATIC ARTERY				PORTAL VEIN				SUPERIOR MESENTERIC VEIN			
	Staging	Restaging			Staging	Restaging			Staging	Restaging			Staging	Restaging			Staging	Restaging		
Case	CE-CT	CE-CT	CE-MRI	DWI-MRI	CE-CT	CE-CT	CE-CT	DWI-MRI	CE-CT	CE-CT	CE-MRI	DWI-MRI	CE-CT	CE-CT	CE-MRI	DWI-MRI	CE-CT	CE-CT	CE-MRI	DWI-MRI
**#1**v	0°	0°	0°	U/A	0°	0°	0°	U/A	>270°	**>270°**	**181−270°**	U/A	>270° (30mm)	>270° (16mm)	U/A	U/A	91−180° (20mm)	U/A	0°	U/A
**#2**v	91−180°	**1−90°**	**0°**	U/A	1−90°	**1−90°**	**0°**	U/A	91−180°	91−180°	91−180°	U/A	91−180° (27mm)	**91−180° (21mm)**	**1−90° (15mm)**	U/A	>270° (45mm)	**91−180° (17mm)**	**0°**	U/A
**#3**v	91−180°	91−180°	91−180°	U/A	0°	0°	0°	U/A	91−180°	**91**−**180°**	**0°**	U/A	>270° (36mm)	91−180° (29mm)	U/A	U/A	91−180° (16mm)	**91−180° (10mm)**	**0°**	U/A
**#4**^^**⌘**^	0°	0°	0°	U/A	0°	0°	0°	U/A	0°	U/A	>270°	U/A	91−180° (20mm)	91−180° (17mm)	>270° (18mm)	U/A	0°	0°	0°	U/A
**#5**v	91−180°	**91**−**180°**	**0°**	U/A	0°	0°	0°	U/A	0°	0°	0°	U/A	0°	0°	0°	U/A	>270° (24mm)	**>270° (23mm)**	**>270° (15mm)**	U/A
**#6x**	0°	0°	0°	0°	0°	0°	0°	U/A	0°	0°	0°	U/A	0°	0°	0°	U/A	91−180° (30mm)	91−180° (20mm)	U/A	U/A
**#7=**	0°	0°	0°	U/A	91c180°	91−180°	91−180°	U/A	0°	0°	0°	U/A	0°	0°	0°	U/A	0°	0°	0°	U/A
**#8**v	181−270°	>270°	>270°	U/A	>270°	>270°	>270°	U/A	>270°	>270°	>270°	U/A	91−180° (13mm)	**91−180° (10mm)**	**91−180° (U/A#)**	U/A	0°	**91−180° (14mm)**	**1−90° (U/A#)**	0°
**#9**v	91−180°	**1**−**90°**	**0°**	U/A	0°	0°	0°	U/A	0°	0°	0°	U/A	0°	0°	0°	U/A	91−180° (25mm)	**1−90° (17mm)**	**0°**	U/A
#10*	0°	0°	0°	0°	0°	0°	0°	0°	0°	0°	0°	0°	0°	0°	0°	0°	0°	0°	0°	0°
**#11^**	0°	0°	0°	U/A	0°	0°	0°	U/A	91−180°	0°	0°	U/A	1−90° (24mm)	0°	1−90° (5mm)	U/A	91−180° (25mm)	91−180° (5mm)	1−90° (10mm)	91−180° (0mm)
**#12**v	1−90°	0°	0°	U/A	0°	0°	0°	U/A	0°	0°	0°	U/A	0°	0°	0°	U/A	>270° (13mm)	**91−180° (15mm)**	**0°**	U/A
**#13**v	0°	**1−90°**	**0°**	U/A	91−180°	**1−90°**	**0°**	U/A	91−180°	**91−180°**	**0°**	U/A	0°	0°	0°	0°	0°	0°	0°	U/A
**#14^**	0°	0°	0°	U/A	0°	0°	0°	U/A	91−180°	91−180°	91−180°	U/A	1−90° (15mm)	1−90° (U/A)	1−90° (12mm)	U/A	0°	0°	0°	U/A
**#15=**	91−180°	91−180°	91−180°	0°	0°	0°	0°	0°	0°	0°	0°	0°	0°	0°	0°	0°	1−90° (20mm)	0°	0°	0°
**#16^**	91−180°	91−180°	91−180°	0°	0°	0°	0°	0°	0°	0°	0°	0°	0°	0°	0°	0°	91−180° (19mm)	1−90° (10mm)	91−180° (16mm)	0°
**#17**v	1−90°	1−90°	1−90°	0°	0°	0°	0°	0°	0°	0°	0°	0°	0°	0°	0°	0°	1−90° (5mm)	**1−90° (7mm)**	**0°**	0°
**#18^**	0°	0°	0°	U/A	0°	0°	0°	U/A	91−180°	**1−90°**	**0°**	U/A	91−180° (21mm)	0°	91−180° (20mm)	U/A	91−180° (21mm)	91−180° (14mm)	91−180° (20mm)	U/A
**#19=**	0°	0°	0°	U/A	0°	0°	0°	U/A	0°	0°	0°	U/A	1−90° (8mm)	0°	0°	U/A	0°	0°	0°	U/A
#20**	0°	0°	U/A	U/A	0°	0°	U/A	U/A	0°	0°	U/A	U/A	1−90° (7mm)	1−90° (6mm)	U/A	U/A	>270° (14mm)	>270° (19mm)	U/A	U/A

*CE-CT*, contrast enhanced computed tomography; *CE-MRI*, contrast enhanced magnetic resonance imaging; *DWI-MRI*, diffusion weighted imaging magnetic resonance imaging; *mm*, millimetres; *U*/*A*, unassessable; *U/A***#**, unassessable by lack of coronal series. **⌘**, staging MRI instead of CE-CT; *, no vascular involvement, so excluded for analysis about radical resection rate; **CE- and DWI-MRI not usable because of major motion artefacts. If patients were used for comparison of vascular involvement on restaging CE-CT versus CE-MRI, the case was marked with one of the following signs that represent the vascular involvement on CE-MRI in comparison to CE-CT: ^, vascular involvement is more on CE-MRI compared to CE-CT; =, vascular involvement is similar on CE-MRI and CE-CT; v, vascular involvement is less on CE-MRI compared to CE-CT; x, vascular involvement cannot be compared by unassessable CE-MRI. The bold numbers indicate that the extent of vascular involvement was less on CE-MRI compared to CE-CT at time of restaging

#### CE-MRI—restaging

CE-MRI was inadequate in one patient because of motion artifacts, so this patient was excluded from these analyses. Comparing CE-MRI and CE-CT scan after induction chemotherapy, the subjectively scored visibility of the tumor delineation (*n* = 15/19, 78.9% *versus n* = 15/19, 78.9%; *p* = 1.000) and vascular tumor involvement (*n* = 12/19, 63.2% *versus n* = 14/19, 73.7%; *p* = 0.727) scored as *moderate/excellent* did not differ significantly. An MRI-halo sign was seen in 52.6% (*n* = 10/19) and a CE-CT-halo sign in 10.5% (*n* = 2/19) (*p* = 0.016) of patients. See Figs. 1c, 2c, and 3c for examples of the halo sign on CE-MRI and Fig. 3b for an example of the halo sign on CE-CT.

At restaging, tumor size tended to be smaller on CE-MRI in comparison to CE-CT scan (26 mm, IQR 20–37 *vs.* 30 mm, IQR 26–42; *p* = 0.073), although this difference was not statistically significant.

Next, only the patients who were diagnosed with vascular involvement at baseline are analyzed (*n* = 18/19, 94.7%), excluding one patient who was staged as LAPC based on multivisceral tumor involvement (i.e., without vascular involvement). In patients with CE-MRI-halo sign (*n* = 10/18, 55.6%), vascular involvement was scored less on CE-MRI in comparison to the extent of vascular involvement on CE-CT scan (*n* = 8/10, 80.0%). In patients without a halo sign on CE-MRI (*n* = 8/18, 44.4%), half of the patients (*n* = 4/8, 50.0%) had more vascular involvement on CE-MRI than on CE-CT scan. Vascular involvement on CE-MRI and CE-CT scan in the remaining four patients was similar (*n* = 2, 25.0%), less on CE-MRI (*n* = 1, 12.5%), or non-assessable on CE-MRI (*n* = 1, 12.5%). In both patients with a halo sign on CE-CT, a halo sign was also seen on CE-MRI. Nevertheless, vascular involvement in both patients was considered less on CE-MRI in comparison CE-CT. See Table 2 for the details about vascular involvement on the restaging imaging.

#### DWI-MRI—restaging

DWI-MRI images were not possible to evaluate in one patient as a consequence of motion artifacts. Therefore, this patient was excluded from the present comparative analyses.

Tumor visibility was scored as excellent (*n* = 4/19, 21.1%), moderate (*n* = 7/19, 36.8%), poor (*n* = 5/19, 26.3%), or invisible (*n* = 3/19, 15.8%) on DWI-MRI. However, assessment of vascular involvement was impossible in nearly all patients, illustrated by the subjective scores *poor* (*n* = 1/19, 5.3%) and *invisible* (*n* = 18/19, 94.7%) in only patients that were diagnosed with vascular involvement at baseline. See Table 3 for details about the tumor delineation and vascular involvement. Comparisons were made with neither CE-MRI nor CE-CT scan because of the general considered poor visibility of DWI-MRI.

**Table 3:** RESTAGING CE-CT versus MRI

	CE-CT				CE-MRI				DWI-MRI			
Case	Tumor delineation	Vascular involvement	Tumor size (mm)	Halo sign	Tumor delineation	Vascular involvement	Tumor size (mm)	Halo sign	Tumor delineation	Vascular involvement	Tumor size (mm)	Halo sign
**#1**	Poor (unusable)	Poor (unusable)	42	No	Poor (unusable)	Moderate	20	Yes	Poor (unusable)	Invisible (unusable)	0	U/A
**#2**	Moderate	Excellent	25	No	Poor (unusable)	Moderate	0	Yes	Excellent	Invisible (unusable)	26	U/A
**#3**	Moderate	Moderate	50	No	Moderate	Poor (unusable)	15	Yes	Excellent	Invisible (unusable)	24	U/A
**#4**	Poor (unusable)	Poor (unusable)	27	No	Moderate	Moderate	32	No	Moderate	Invisible (unusable)	18	U/A
**#5**	Moderate	Moderate	26	No	Moderate	Moderate	18	Yes	Invisible (unusable)	Invisible (unusable)	0	U/A
**#6**	Excellent	Moderate	34	No	Excellent	Poor (unusable)	37	No	Poor (unusable)	Invisible (unusable)	35	No
**#7**	Excellent	Moderate	45	No	Moderate	Moderate	41	No	Invisible (unusable)	Invisible (unusable)	0	U/A
**#8**	Moderate	Moderate	55	No	Excellent	Moderate	53	No	Invisible (unusable)	Invisible (unusable)	0	U/A
**#9**	Moderate	Moderate	29	Yes	Moderate	Moderate	17	Yes	Poor (unusable)	Invisible (unusable)	18	Yes
#10*	Excellent	Excellent	90	No	Excellent	Excellent	88	No	Moderate	Invisible (unusable)	88	No
**#11**	Moderate	Poor (unusable)	31	No	Moderate	Poor (unusable)	24	Yes	Moderate	Poor (unusable)	19	No
**#12**	Excellent	Excellent	28	Yes	Excellent	Moderate	22	Yes	Moderate	Invisible (unusable)	30	U/A
**#13**	Moderate	Moderate	30	No	Moderate	Moderate	41	Yes	Poor (unusable)	Invisible (unusable)	36	U/A
**#14**	Poor (unusable)	Poor (unusable)	26	No	Poor (unusable)	Poor (unusable)	21	No	Moderate	Invisible (unusable)	31	No
**#15**	Moderate	Moderate	33	No	Excellent	Moderate	28	No	Excellent	Invisible (unusable)	22	No
**#16**	Moderate	Moderate	32	No	Moderate	Poor (unusable)	26	No	Poor (unusable)	Invisible (unusable)	0	No
**#17**	Excellent	Moderate	26	No	Poor (unusable)	Poor (unusable)	23	Yes	Moderate	Invisible (unusable)	32	No
**#18**	Moderate	Moderate	24	U/A	Moderate	Poor (unusable)	29	No	Excellent	Invisible (unusable)	30	U/A
**#19**	Invisible (unusable)	Invisible (unusable)	0	No	Excellent	Excellent	28	Yes	Moderate	Invisible (unusable)	28	U/A
#20**	Excellent	Moderate	17	No	-	-	-	-	-	-	-	-

*CE-CT*, contrast enhanced computed tomography; *CE-MRI*, contrast enhanced magnetic resonance imaging; *DWI*-*MRI*, diffusion weighted imaging magnetic resonance imaging; mm, millimetres; *U*/*A*, unassessable; *, no vascular involvement, so excluded for analysis about radical resection rate; **CE- and DWI-MRI not usable because of major motion artefacts

**Table 4.**
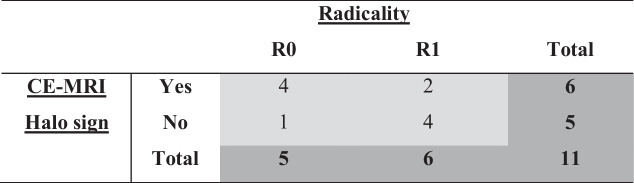
CE-MRI-halo sign in patients undergoing resection—radicality*

*CE-MRI*, contrast-enhanced magnetic resonance imaging. *n = 11 patients with vascular involvement at time of diagnosis and adequate MRI.

See Fig. 1d for a relative useful DWI-MRI. However, the low signal of the superior mesenteric vein limits the assessment of the absence/presence of a halo sign. See Fig. 2d for a clinically useless DWI-MRI whereby the tumor delineation is poorly visible and vascular involvement is invisible.

### Clinical outcome

#### Surgical and pathological outcome

All selected patients were surgically explored with the intention of resection, of whom 60% finally underwent a resection (*n* = 12/20), including pancreatoduodenectomy (*n* = 9, 75.0%), distal pancreatectomy (*n* = 2, 16.7%), and total pancreatectomy (*n* = 1, 8.3%). Vascular resection was required in 9 patients (75.0%), including portomesenteric venous (*n* = 8, 66.7%) and celiac axis resection (*n* = 1, 8.3%). Multivisceral resection was required in one patient (8.3%). Surgical resection was not feasible in the remaining 8 patients, because of metastatic disease (*n* = 4, 20.0%) or extensive local regional tumor ingrowth from peripancreatic vasculature (*n* = 4, 20.0%). From this latter group, all patients were treated with radiofrequency ablation in the setting of a clinical trial [38]. See Supplementary Digital Content 2 for surgical and pathology outcome.

From the 18 patients with vascular involvement at time of diagnosis and adequate MRI (excluding the patient without vascular involvement and the patient with motion artifacts), the (R0) resection rates are described. Six out of 10 patients (60.0%) with a halo sign on CE-MRI underwent a resection, compared to 5 out of 8 patients (62.5%) without a halo sign (*p* = 1.000). Four out of 6 patients (66.7%) with a CE-MRI-halo sign who underwent a resection had an R0 resection, compared to 1 out of 5 patients (20.0%) who underwent a resection without a halo sign on CE-MRI (*p* = 0.242).

In the patients who underwent a resection, the CE-MRI-halo sign to predict an R0 resection had a sensitivity, specificity, positive predictive value, and negative predictive value of 80%, 66.7%, 66.7%, and 66.7%. See Table 4 for the cross table.

## Discussion

The findings of this first prospective pilot study, investigating the added value of CE-MRI in assessing tumor response in LAPC in addition to CE-CT, suggest less tumor involvement and smaller tumor size after induction chemotherapy on CE-MRI by the more frequently seen halo sign on CE-MRI (53%) compared to CE-CT (11%). Patients with a CE-MRI-halo sign appeared to have a non-significant higher rate of R0 resection in comparison to patients without an CE-MRI-halo sign, but this should be seen as purely hypothesis generating and should be confirmed in larger studies. In contrast, vascular involvement was mainly not assessable on DWI-MRI and thus may not have clinical utility for local, morphological staging purposes.

MRI is mentioned as a potential functional imaging modality (e.g., DWI-MRI) to predict oncological outcomes (e.g., histopathological response, R0 resection, disease-free, and overall survival) after chemo(radio)therapy in patients with pancreatic cancer [16, 23, 39]. On the other hand, the role of MRI as morphological imaging modality in these patients is unclear. A recent review paper from the Society of Abdominal Radiology stated that the limitations of morphological imaging (both CT and MRI scan) after chemo(radio)therapy for PDAC are well established [23]. Of note, this view on MRI seems to be based on a meta-analysis [40] from the era where preoperative chemo(radio)therapy was not widely implemented yet. To our best knowledge, in-depth literature about the value of MRI for morphological restaging after chemo(radio)therapy for PDAC is still lacking.

The present study suggests that CE-MRI might have added value when restaging LAPC following induction chemotherapy, next to CE-CT. The presence or absence of a halo sign on CE-MRI could discriminate between vital tumor tissue and fibrosis after induction therapy although further larger prospective studies are needed to prove this suggestion. This hypothesis is carefully strengthened by differences in vascular involvement between CE-CT scan and CE-MRI, where vascular involvement seemed generally less on MRI in the presence of an MRI-halo sign, whereas the opposite appeared to be true in the absence of an MRI-halo sign (i.e., increased vascular involvement compared with CT scan). The finding that the halo sign was more frequently seen on CE-MRI than on CE-CT may suggest that MRI might be more sensitive to assess post-chemotherapy resectability, but this needs to be proven in larger studies.

This study confirms that DWI-MRI is insufficient for morphological staging since tumor delineation and vascular tumor involvement were hardly assessable. This may not come as a surprise, given the generally low spatial resolution of this modality. Interestingly, also the borders of the tumors were difficult to distinguish from the normal pancreas parenchyma since the pancreas often demonstrated diffusion restriction. This phenomenon was also observed in one of our previous series, investigating different models like DWI-MRI models in patients with (borderline) resectable pancreatic cancer who were treated with neoadjuvant gemcitabine plus hypo-fractionated radiation [21]. Possibly, this is a result of chemotherapy [41], but this cannot be confirmed with the present study because no MRI scan was made before chemotherapy.

Whereas the current results suggest the potential value of CE-MRI for morphological response evaluation after induction chemotherapy, the benefits of CT scan over MRI comprise a higher contrast resolution (that is key for adequate tumor delineation), shorter scan time (so fewer motion artifacts and less burden for patients), and lower costs [18, 23]. Taking this into account, optimization of CE-CT interpretation (especially regarding arterial involvement) could be helpful [19], next to investigating the additional or alternative role of other imaging modalities such as CE-MRI. In recent years, various modified criteria and composite scores are proposed, aiming to optimize the predictive value of CT scan after preoperative therapy. Noda and colleagues designed a composite score based on the presence and characteristics of arterial involvement and resectability status before and after preoperative therapy [42]. This score was correlated with both R0 and overall survival. However, this score was biased since it was developed in a cohort that underwent surgical resection [42]. In addition, Yang et al. investigated the diagnostic accuracy of different CT criteria (National Comprehensive Cancer Network [NCCN] *versus* modified criteria) based on published literature [15]. Modified resectability criteria involved more nuances in the assessment of vascular involvement, such as changes in perivascular tissue compared to the pretreatment situation. Meta-analysis demonstrated a higher diagnostic accuracy to predict R0 resection when using the modified criteria (0.78 [95% CI 0.74–0.82] *versus* 0.67 [95% CI 0.63–0.71]) [15]. Ahmed and colleagues showed that more detailed criteria involving the degree and length of involvement and vessel deformation are reproducible and sufficient predictors for R0 resection [31, 43]. In addition to such modified criteria, the change of tumor attenuation on CE-CT scan may be useful to predict R0 resection [44] as well as the tumor homogeneity that seems to correlate with disease-free and overall survival [45]. Further nuance in resectability from advanced PDAC with major arterial tumor involvement based on CT imaging can be made with the presence of a string versus halo sign, possibly differentiating between the presence and absence of vessel wall invasion [26, 27]. In addition, the halo sign on CE-CT as sign of absent vascular invasion has also been described for venous involvement (25). In the future, the extraction of radiomic features could further improve the judgment of resectability after chemo(radio)therapy [46, 47].

The often-overestimated vascular tumor involvement on CE-CT scans after chemotherapy might, to a yet undefined extent, be more accurately assessed with (additional) CE-MRI. If this will be confirmed by more robust studies, CE-MRI could possibly improve the anatomical restaging of LAPC after induction chemotherapy, allowing for better selection for oncological and surgical treatment. Further prospective observational studies are required to investigate the diagnostic accuracy and clinical value of CE-MRI in comparison/next to CE-CT scan to predict R0 resection and to investigate the meaning of a halo sign by involving the histopathological ground truth. Interestingly, an association of CE-MRI-halo sign and tumor marker patterns may be valuable in the development of prognostic models. In addition, other predictors for resectability on CE-MRI have to be investigated (e.g., tumor shrinkage and [changes in] contrast enhancement) as well as the predictive value of DWI-MRI with ADC quantification. Until the role of CE-MRI is validated, the above-mentioned CT criteria might support the anatomical restaging with CE-CT scan. Additionally, patient selection could be further improved by implementing the use of PET combined with CT or MRI before and after induction chemo(radio)therapy for local response evaluation [8, 39, 48] and intraoperative ultrasonography during an exploratory laparotomy to assess the (extent of) vascular tumor involvement [49, 50].

The present study should be interpreted in the light of several limitations and strengths. First, no MRI was performed before induction chemotherapy, so the findings cannot be related to the baseline situation. Second, the sample size is small since this was a pilot study, giving limited value to the calculated diagnostic performance parameters. Since only 2 patients had a CE-CT-halo sign, calculating the diagnostic performance and a subsequent comparison with the diagnostic performance of the CE-MRI-halo sign was considered not reliable. In addition, the diagnostic performance of the DWI-MRI was not calculated, because of the evident lack of value for morphological restaging. Third, the interobserver variability was not assessed since the imaging was immediately scored in consensus. However, as the CE-MRI-halo sign is a new phenomenon, our radiologists chose to work in consensus in order to learn how to interpret the sign and reducing the impact of confounders as a consequence of anatomy-based categorization of vascular involvement. To the best of our knowledge, this is the first prospective study that investigated the role of MRI for local morphological restaging in patients with LAPC patients after induction chemotherapy.

## Conclusions

This prospective pilot study suggests that CE-MRI might be helpful to determine the tumor size and vascular involvement of LAPC after induction chemotherapy including the “CE-MRI-halo sign” for tumor response that possibly enables better assessment of the actual vascular involvement with vital tumor *versus* fibrosis. However, this finding should be seen as hypothesis generating rather than absolute proof of value of the CE-MRI-halo sign, which requires larger prospective studies.

## Supplementary information

Below is the link to the electronic supplementary material.

Supplementary file1 (DOCX 18 KB)

Supplementary file2 (DOCX 86 KB)

## Data Availability

The data used to support the findings of this study are available from the corresponding author upon reasonable request.
