# Supplementary material for: Added value of 3T MRI and the MRI-halo sign in assessing resectability of locally advanced pancreatic cancer following induction chemotherapy (IMAGE-MRI): prospective pilot study
Source: Langenbecks Arch Surg. 2022 Oct 15;407(8):3487–99. doi: 10.1007/s00423-022-02653-y (PMC9722850; doi:10.1007/s00423-022-02653-y)
Supplement: Supplementary file 1 — Supplementary file1 (DOCX 18 KB) [file 423_2022_2653_MOESM1_ESM.docx]

**SUPPLEMENTARY DIGITAL CONTENT 1.** IMAGING – PROCEDURAL DETAILS

*CT imaging*

All pre- and post-chemotherapy CT scans consisted of a chest and abdominal CT scan according to a biphasic protocol with a late arterial phase (35-40 seconds after intravenous contrast injection) and a portal venous phase (60-70 seconds after intravenous contrast injection). CT scans were performed on a Siemens Sensation 64-slice CT-scanner (Siemens, Erlagen, Germany) after injection of 1.5-2.0 ml/kg (with a maximum of 120 ml) iopromide (Bayer AG, Berlin, Germany) contrast medium at an injection rate of 3.5 ml/s using an automatic injector through an antecubital vein and followed by 40 ml of saline solution chase with the same flow rate. Other scanning parameters were reconstruction slice thickness 2 mm for the late arterial phase (120 kV, 120 mAs, pitch 0,9) and 3 mm for the portal-venous phase images (120 kV, 160 mAs, pitch 0,9). Reconstructions were made in the transverse, sagittal and coronal planes. After chemotherapy, the CT examinations were repeated with the same protocol.

Exceptions on the above-mentioned protocol were the CT scans of three patients who were made in another hospital from the Dutch Pancreatic Cancer Group [33]. Four CT scans from two patients were made at a Philips ICT (256 slice, Philips, Best, The Netherlands), using contrast medium iomeprol (bracco). Doses of contrast and reconstruction parameters were identical to the above-mentioned protocol. Furthermore, another patient underwent a CT scan, using the Siemens Somaton Definition 2x129 slice dual energy Flash scanner. The contrast medium concerned ioversol 350 (98 ml contrast with an injection speed of 4,5 ml/s, followed by 20 ml of saline solution). Slice thickness was 3mm and reconstructions were made in the earlier mentioned three planes. Late arterial phase (pancreatic phase) and portal venous phase images were used for identification and delineation of the pancreatic tumor and visibility of vascular involvement.

*MRI imaging*

All MRI scans were acquired on a 3T MRI scanner (Ingenia, Philips, Best, The Netherlands). Before MRI scanning, patients were administered pineapple juice as an oral contrast-agent to suppress fluid T2 signal from the duodenum. Hyoscine bromide (Buscopan, Boehringer, Ingelheim, Germany; 20 mg i.v.) was administered directly before the first DWI acquisition in each scan session to minimize bowel motion.

First a multi-slice 2D T2-weighted turbo spin echo was acquired in three breath holds of 15 – 17 seconds, with repetition time / echo time (TR/TE: 712,3/80 ms; field of view (FOV): 448x320 mm^2^, acquisition voxel size: 2x2 mm^2^, reconstruction voxel size 1x1 mm^2^, slice thickness 5 mm with no gap; 40 slices, parallel imaging factor 2, echotrain 56, refocusing angle 120°).

Next axial multi-slice 2D diffusion-weighted echo-planar imaging MRI with fat suppression were acquired during free-breathing (TR/TE time: 2166/62.7 ms, FOV: 432x320 mm^2^, acquired voxel size: 2.5x 2.5, reconstructed voxel size: 1.1x1.1 mm^2^, 5 mm slice thickness with 1 mm slice gaps, 34 slices, parallel imaging factor 3, partial Fourier factor 0.64 b-values: 0, 150 and 800 seconds/mm^2^, with 6, 6 and 12 averages, respectively. From the diffusion MRI series, ADC maps were computed. In addition, a 3D T1-weighted multi-echo (3 echoes) spoiled gradient echo (T1W GE) with three-point Dixon reconstruction was performed before and six times (30, 80, 120, 180, 240, 300s) after contrast injection, each in single breath holds of 15 - 17 seconds per slab (repetition time: 6.0 ms, echo time: 1.45/2.51/3.67 ms, FOV: 402x275x120 mm^2^ acquired voxel size: 1.2x1.2x2.4 mm^3^, reconstructed voxel size: 0.93x0.93x1.2 mm^3^, flip angle: 20°, parallel imaging 2.4/1.5). For contrast-enhanced imaging, gadolinium i.v. was used (Gadovist, Bayer) with a dose of 0.1 mmol/kilogram.

For the evaluation of the pancreatic tumor and vascular involvement, the 30 and 80 seconds post-contrast injection phases were used. Similarly, the high B-value (B800) and ADC images were selected when using the diffusion-weighted sequences.
