# Supplementary material for: Added value of 3T MRI and the MRI-halo sign in assessing resectability of locally advanced pancreatic cancer following induction chemotherapy (IMAGE-MRI): prospective pilot study
Source: Langenbecks Arch Surg. 2022 Oct 15;407(8):3487–99. doi: 10.1007/s00423-022-02653-y (PMC9722850; doi:10.1007/s00423-022-02653-y)
Supplement: Supplementary file 2 — Supplementary file2 (DOCX 86 KB) [file 423_2022_2653_MOESM2_ESM.docx]

| **SUPPLEMENTARY DIGITAL CONTENT 2.** SURGERY AND PATHOLOGY | | | | | | | | | | |
| --- | --- | --- | --- | --- | --- | --- | --- | --- | --- | --- |
|  | **SURGERY** | |  | **PATHOLOGY** | | | | | | |
| **Case** | **Resection** | **Vascular resection^#^** |  | **R status** | **Tumor size (mm)** | **Differentiation** | **Lymphovascular invasion** | **Perineural invasion** | **Positive / total lymph nodes (*n*)** | **Distant metastasis** |
| **#1** | No, RFA | - |  | - | - | - | - | - | - | No |
| **#2** | PD | PV-confluence |  | R1 (neck margin) | 30 | Well | No | Yes | 0/17 | No |
| **#3** | PD | SMV |  | R1 (SMA margin) | 30 | Poor | No | No | 2/17 | No |
| **#4** | TP | PV |  | R0 | Unknown | Poor | No | No | 0/31 | No |
| **#5** | No, RFA | - |  | - | - | - | - | - | - | No |
| **#6** | PD | SMV |  | R1 (SMV margin) | 45 | Poor | Yes | Yes | 2/10 | No |
| **#7** | DP | Celiac axis |  | R1 (anterior & posterior margin) | 50 | Poor | Yes | Yes | 5/29 | No |
| **#8** | No, RFA | - |  | - | - | - | - | - | - | No |
| **#9** | PD | 1st branch jejunal vein |  | R0 | 30 | Moderate | Yes | No | 16/21 | No |
| **#10** | No | - |  | - | - | - | - | - | - | Yes |
| **#11** | No, RFA | - |  | - | - | - | - | - | - | Yes |
| **#12** | PD | No |  | R0 | 30 | Well | No | Yes | 1/8 | No |
| **#13** | No | - |  | - | - | - | - | - | - | Yes |
| **#14** | No | - |  | - | - | - | - | - | - | No |
| **#15** | No | - |  | - | - | - | - | - | - | Yes |
| **#16** | PD | No |  | R1 (SMA margin) | 32 | Unknown | No | Yes | 0/28 | No |
| **#17** | PD | SMV |  | R0 | 29 | Unknown | Yes | Yes | 3/18 | No |
| **#18** | PD | SMV |  | R1 (neck margin) | 50 | Unknown | Yes | Yes | 1/19 | No |
| **#19** | DP | No |  | R0 (anterior margin R1) | 33 | Moderate | Yes | Yes | 1/2 | No |
| **#20** | PD | Confluence-SMV |  | R0 | 16 | Unknown | Yes | No | 0/13 | No |
| #, resection of portomesenteric venous axis, celiac axis, any hepatic artery, and/or superior mesenteric artery; *mm*, millimeters; *n,* number of lymph nodes; *RFA*, radiofrequency ablation; *PD*, pancreatoduodenectomy; *TP*, total pancreatectomy; *DP*, distal pancreatectomy; *SMA*, superior mesenteric artery, *SMV*, superior mesenteric vein; *, no vascular involvement, so excluded for analysis about radical resection rate; **CE- and DWI-MRI not usable because of major motion artefacts. | | | | | | | | | | |
